# News and Notes

**Published:** 1903-01

**Authors:** 


### *NEWS AND NOTES.*

(We are indebted to the *Medical Age* for many of these notes.)

---

THERE are at present over a dozen lepers under treatment in the St. Louis Hospi al, Paris.

---

DURING the month of October seven cases of plague occurred in San Francisco, all ending fatally.

---

DR. JACQUES LOEB, of Chicago, has been appointed Professor of Physiology in the University of California.

---

THE second meeting of the Latin-American Medical Congress will be held in April, 1904, at Buenos Ayres.

---

THE citizens of Calumet, Michigan, have erected a public hospital sufficient to accommodate twenty-five patients.

---

CHOLERA has reappeared among the men in the Fifth Infantry stationed at Manila. A number have already died, and many others are seriously ill.

---

A NEW form of death certificate has been adopted by the New York State Board of Health. It follows closely the one prepared by the Committee on Demography of the American Public Health Association.

---

IN the event of Rush Medical College raising the sum of $1,000,000 before July 1, 1903, the trustees of the University of Chicago have agreed to receive the medical school as a part of the University of Chicago.

THE Pan-American Sanitary Congress met in Washington on December 2. The object of the conference was to definitely discuss some practicably universal plans for the prevention and suppression of yellow fever and other plagues in Pan-American ports.

---

A FRENCH investigator has recently come to the conclusion, says *American Medicine*, that the brains of military men give out most quickly. He states that out of every 100,000 military men 199 become hopeless lunatics. Of the liberal professions, artists are the first to succumb to brain strain, next lawyers, followed at some distance by doctors, clergymen, literary men and civil servants. Striking an average of this group, 177 out of each 100,000 become insane.

---

WE have received a pamphlet published by the Chas. Roome Parmele Co., entitled Diabetes Mellitus—Its Treatment and Cure—Clinical Reports by Drs. Stucky, Dixon, Hickey, Haines, Bishop and others. The reports are highly laudatory of the bromide of gold and arsenic, known as Arsenauro. We might add from our own experience the report of an apparently complete cure of diabetes complicated with gangrenous ulcer of the leg. The patient after three years is in good health.

---

ACCORDING to *Science*, Dr. Swale Vincent, lecturer on histology at the University College, Cardiff, and formerly assistant professor of physiology at University College, London, who has already made numerous contributions to the literature of the ductless glands, has been appointed to the research scholarship for the study of the thymus and other ductless glands recently established in England by Mr. J. Francis Mason. Mr. Mason has also made a donation of £200 to the laboratory of the Edinburgh Royal College of Physicians to enable the medical superintendent, Dr. Noel Paton, to carry out combined research on the ductless glands. Professor Ribbert, of Marburg, has been appointed director of the Pathological Institute at Göttingen in succession to Professor Orth, who succeeded the late Professor Virchow as Professor of Pathology in Berlin.

THE National Association for the Study of Epilepsy held its annual meeting at the New York Academy of Medicine on November 5 under the presidency of Dr. Frederick Peterson, of New York. In the course of his address Dr. Peterson said that the Craig Colony of New York can care for 1,126 patients, with property valued at $666,500. Eighty of the inmates attend school. The cost is $3.15 per week. There are at least 500 more, according to the census, requiring such care. Massachusetts has expended $85,900 on her colony for the epileptic, and of the 1,440 epileptics of that State she is caring for 440, at a cost of $4.50 a week; 24 are in school. New Jersey, with 1,500 needing care, as yet has but 75 in her colony, at a cost of $4.50 a week. Dr. Wharton Sinkler, of Philadelphia, was elected the next president, and Dr. Spratling, of the Craig Colony, was reelected secretary.—*New York Medical Journal.*

---

THE Army Medical School was, according to the *New York Medical Journal,* opened in the Army Medical Museum at Washington on November 10 with a class of forty student-officers in attendance. For the current session, the seventh, the faculty is composed of the following officers in the United States Army Medical Corps: Colonel Calvin De Witt, assistant surgeon-general, president of the faculty and professor of military medicine; Major Walter Reed, surgeon, professor of clinical microscopy; Major Louis A. La Garde, surgeon, professor of ophthalmology and lecturer on "Duties of Military Officers"; Major W. C. Borden, surgeon and professor of military surgery; Major Walter D. McGraw, surgeon and professor of military hygiene; Captain Frederick P. Reynolds, assistant surgeon, instructor in first aid hospital corps drill and field hospital administration; Captain Carl R. Darnall, assistant surgeon, secretary of the faculty and instructor in sanitary chemistry. In addition to the instructions mentioned, lectures on military law are given by Brigadier-General George B. Davis, judge-advocate general, and lectures on those tropical diseases with which our troops have been afflicted in the Philippines will be given by First Lieutenant Joseph H. Ford, assistant surgeon.

